# Variational Hilbert Quantitative Phase Imaging

**DOI:** 10.1038/s41598-020-69717-1

**Published:** 2020-08-18

**Authors:** Maciej Trusiak, Maria Cywinska, Vicente Mico, Jose-Angel Picazo-Bueno, Chao Zuo, Piotr Zdankowski, Krzysztof Patorski

**Affiliations:** 1 Institute of Micromechanics and Photonics, Warsaw University of Technology, 8 Sw. A. Boboli St., 02-525 Warsaw, Poland; 2 Departamento de Óptica y de Optometría y Ciencias de la Visión, Facultad de Física, Universitat de Valencia, C/Doctor Moliner 50, 46100 Burjassot, Spain; 3 Jiangsu Key Laboratory of Spectral Imaging and Intelligence Sense, Nanjing University of Science and Technology, Nanjing, 210094 Jiangsu China

**Keywords:** Imaging and sensing, Optical metrology, Interference microscopy, Applied optics

## Abstract

Utilizing the refractive index as the endogenous contrast agent to noninvasively study transparent cells is a working principle of emerging quantitative phase imaging (QPI). In this contribution, we propose the Variational Hilbert Quantitative Phase Imaging (VHQPI)—end-to-end purely computational add-on module able to improve performance of a QPI-unit without hardware modifications. The VHQPI, deploying unique merger of tailored variational image decomposition and enhanced Hilbert spiral transform, adaptively provides high quality map of sample-induced phase delay, accepting particularly wide range of input single-shot interferograms (from off-axis to quasi on-axis configurations). It especially promotes high space-bandwidth-product QPI configurations alleviating the spectral overlapping problem. The VHQPI is tailored to deal with cumbersome interference patterns related to detailed locally varying biological objects with possibly high dynamic range of phase and relatively low carrier. In post-processing, the slowly varying phase-term associated with the instrumental optical aberrations is eliminated upon variational analysis to further boost the phase-imaging capabilities. The VHQPI is thoroughly studied employing numerical simulations and successfully validated using static and dynamic cells phase-analysis. It compares favorably with other single-shot phase reconstruction techniques based on the Fourier and Hilbert–Huang transforms, both in terms of visual inspection and quantitative evaluation, potentially opening up new possibilities in QPI.

## Introduction

Optical imaging is a central aspect of biological research, biomedical examination, and medical diagnosis. Optical microscope is indispensable in biological and medical research facilitating a “seeing is believing” paradigm^1^. Despite its rich history and well-established position, significant efforts are contemporarily put on developing new imaging modalities enabling enhanced resolution, contrast, depth, speed and information content. Among a suite of modern microscopy techniques, quantitative phase imaging (QPI)^2–4^ stands out as a vividly blossoming label-free approach. It provides unique means for imaging cells and tissues merging beneficial features identified with microscopy^1^, interferometry and holography^5^, and numerical computations. Using refractive index as the endogenous contrast agent^6,7^ QPI numerically converts recorded interference pattern into a nanoscale-precise subcellular-specific map of optical delay introduced by examined transparent specimen^7–9^. This non-phototoxic non-destructive imaging technique brings biology and metrology closer as it generates quantitative maps of analyzed live bio-structure (related to cell mass, volume, surface area, and their evolutions in time). Therefore, QPI enables upgrading phase-contrast based^6^ visualization of the sample to stain-free non-invasive measurement of sample-induced optical phase delay (related to refractive index and/or thickness variations). Impressive details can be imaged, e.g., via super-resolution approaches^10,11^ even in live cells without photo-damage^10^.

Application oriented research flourished recently providing outstanding and exciting solutions, e.g.,

- in neuroscience: digital holographic microscopy DHM^12, 13^, spatial light interference microscopy SLIM^14, 15^, optical diffraction tomography ODT^11,16^;
- in cell/tissue biology: ODT (stem cells)^17^, SLIM^14^, Hilbert phase microscopy HPM^18^, transport of intensity equation^19^, Fourier ptychography^20^, quadriwave lateral shearing interferometry QLSI^21,22^, gradient light interference microscopy GLIM^23^, Hilbert–Huang phase microscopy H2PM^24^, flow cytometry^25^;
- in cancer diagnosis: DHM^26^, SLIM^27^, diffraction phase microscopy DPM^28,29^, to name only a representative number of approaches.

Phase stability can be enhanced using common-path strategies^30^ while partial coherence is used to decrease speckle noise^31^. Separate interesting group of methods comprises lens-free compact optical setups operating in in-line Gabor holography regime with numerical reconstruction of the sample structure^32^ and on-chip solutions^33^. Tomographic techniques enable three-dimensional (3D) QPI employing either full projection^34^ or limited angle approaches based on regularized reconstruction^35^.

QPI solutions based on digital holographic microscopy (DHM, ODT) and interferometry (e.g., SLIM, GLIM, QLSI, DPM, HPM, H2PM) are full-field optical techniques relying on encoding complex amplitude in an intensity pattern (modulated in phase and amplitude). The main challenge is then to decode (demodulate, retrieve, reconstruct) the phase map of interest from the recorded intensity distribution. Presented contribution focuses on the QPI approaches generating and demodulating fringe patterns (i.e., DHM, ODT, HPM, QLSI, H2PM) rather than the ones based on iterative multi-frame phase retrieval i.e., Fourier ptychography^20^ or transport of intensity^19^. Interferogram containing the required object information is constructed upon coherent superimposition (interference) of object and reference beams. Two-beam interference pattern consists of a sum of three fundamental intensity components (Eq. 1): background (*a*, incoherent sum of intensities I_1_ and I_2_ of interfering beams), noise (*n*, uncorrelated and/or structured) and coherent interference fringes comprised by a cosine function modulated in phase (θ) and amplitude (*b*, 2(I_1_I_2_)^1/2^):

1 $$I = I_{1} + I_{2} + 2\sqrt {I_{1} I_{2} } \cos (\theta ) + n = a + b\cos (\theta ) + n.$$

Phase distribution of interest is encoded in the local shape of fringes—period and orientation variations. Therefore, extremely important step of each fringe-based full-field QPI method encompasses interferogram phase demodulation understood as phase map decoding from recorded intensity distribution. Quality and robustness of this numerical operator (phase demodulator) directly defines the accuracy and capabilities of each QPI unit while imposing important limitations. Hence, one tends to upgrade numerical processing to boost the overall quality of the QPI method/device/examination.

There are two main QPI architectures with respect to the phase demodulation strategy employed: on-axis layouts with temporal phase shifting based phase demodulation^36^ and off-axis layouts with Fourier transform (FT) based phase retrieval^37^. The on/off axis term refers to the inclination angle between object and reference beams—it is significantly increased in off-axis case to produce high carrier spatial frequency and spectrally separate otherwise overlapped cross-correlation object terms from auto-correlation peak to facilitate its successful Fourier-filtering. Both approaches are somewhat promoted and penalized: on-axis recording with phase shifting (e.g., SLIM and GLIM) despite being the most accurate and well optimized in terms of space-bandwidth product has limited time resolution due to the need of interferogram phase-shifting sequence recording. Off-axis configuration aided by Fourier transform can quickly analyze dynamic transient events in a single-shot manner (phase is retrieved from single interferogram) but requires sufficiently high carrier spatial frequency limiting space-bandwidth product and imposing constraints on phase details of biostructure images (generally, object should be low-pass banded with respect to carrier frequency).

It is worth emphasizing that third configuration emerged recently, namely the slightly off-axis regime. It attempts the space-bandwidth product optimization by means of full spectral separation of conjugated object lobes, while leaving the autocorrelation term partially overlapped with information carrying cross-correlation terms^38–52^. Slightly off-axis phase demodulation is presently facilitated mainly by imposing restrictions on object and reference beams^38–40^, utilizing subtraction of two images^41–47^, employing two wavelengths^48^, or basing on the 1D limited processing^49–52^, however. In this contribution we are focusing on accurate bi-dimensional single-shot approaches as ability to study full-field dynamic events is fundamental in biomedicine. Single-shot slightly-off axis methods with numerical operators require either constrains on the usable field of view^43,47^, object and reference beam intensities^38,39^ and/or complicated optimization^40^, or are limited to image 1D scanning^49–52^. Nonetheless, recently a very elegant approach employing Kramers–Kronig relations was introduced to the QPI and digital holographic imaging in general^53^. Although it is able to significantly augment the space-bandwidth product of full complex field recovery, and therefore expand capabilities of nearly all QPI units, it requires separation of interferogram’s cross-correlation terms in the Fourier domain. Quasi (and fully) on-axis configurations, where both conjugated object (cross-correlation) terms spectrally overlap with each other and the autocorrelation term, are very challenging, hence constitute an exciting direction for possible improvements. It is to be emphasized that single-shot, single-wavelength, full-FOV and beam intensity constraint-free analysis of such spectrum-overlapped carrier-less interferograms composes a very cumbersome and important problem, yet to be addressed in the QPI field. Versatile two-dimensional approach able to efficiently analyze single interferogram regardless of its characteristics (noise, low contrast, strong local fringe shape variations etc.) is still expected. It would enable to somewhat merge on-axis and off-axis regimes and capitalize on the advantages of both approaches simultaneously.

In this virtue, we propose the Variational Hilbert Quantitative Phase Imaging (VHQPI) method—end-to-end purely numerical add-on module improving the QPI performance without any hardware modifications. It adaptively and automatically alleviates the overlapped-spectrum problem employing advanced variational preprocessing of interferograms and enhanced Hilbert spiral transform based phase demodulation. The VHQPI accepts as input extremely wide range of single-shot interferograms, in terms of quality, carrier frequency and object spectrum, providing detail-rich phase map as output in all-in-one operation manner.

Paper is organized as follows. “Proposed numerical scheme description” introduces VHQPI regarding its processing path and analyzes numerical examples. “Numerical evaluation using synthetic interferograms” evaluates the VHQPI using synthetic holograms. “Experimental evaluation” validates the proposed VHQPI method considering different types of bio-samples and the last section concludes the paper.

## Proposed numerical scheme description

In this section we will focus on describing step-by-step the proposed new end-to-end processing path for QPI.

### Interferogram initial filtering

In the first step, single interferogram is decomposed into three terms: (1) low-frequency background term (associated with the incoherent sum of intensities of interfering beams), (2) high-frequency noise (not correlated with the fringes) and (3) coherent interference term of interest (fringes). This image domain based dissection of interferogram delivers three fundamental components using modified unsupervised Variational Image Decomposition (uVID) approach^54–57^. It is based on the notion of the classical VID^58–60^ to extract noise, structure (background) and texture (fringes) of the image with emphasis put on several essential incorporated advancements^54–57^: (1) noise is removed with remarkable efficiency using block-matching 3D algorithm^61,62^; (2) structure-texture differentiation is performed utilizing modified Chambolle projection^63^ algorithm with automated stopping criterion to set the number of projections in a data-driven interferogram-specific adaptive manner^57^; (3) there is no need to pre-set any parameter value. All introduced advancements of our fringe pattern pre-processing algorithm are thoroughly described in^57^. Here, we propose to use it for QPI performance improvement.

The important fact to comprehend is that there are three different parameters (regularization constant *µ,* projection step* τ* and number of iterations *NI*) to be set in order to get the variational image decomposition result. Analyses in^54–57^ provided detailed corroboration of the versatility and efficiency of the algorithm employing fixed, but versatile, values for two first parameters (100 and 0.25, respectively) and automatic optimization scheme for finding the best number of iterations (*NI*; method proposed in^57^ using tolerance calculation has been proven to work efficiently for majority of interferograms). All mentioned features make the modified unsupervised VID (uVID) approach unique and extremely versatile. Essentially, any interferogram containing distinguishable fringes (from few ones to Nyquist limit) can be successfully filtered in the automatic manner^57^ regardless the phase map characteristics (fringe shape and density variations), spectrum overlapping (carrier-free patterns) and the noise level (reasonably high with respect to the signal level). The main purpose of the uVID method is to accurately remove background and minimize noise within the single fringe pattern.

### Filtered interferogram phase demodulation

**Figure 1:**
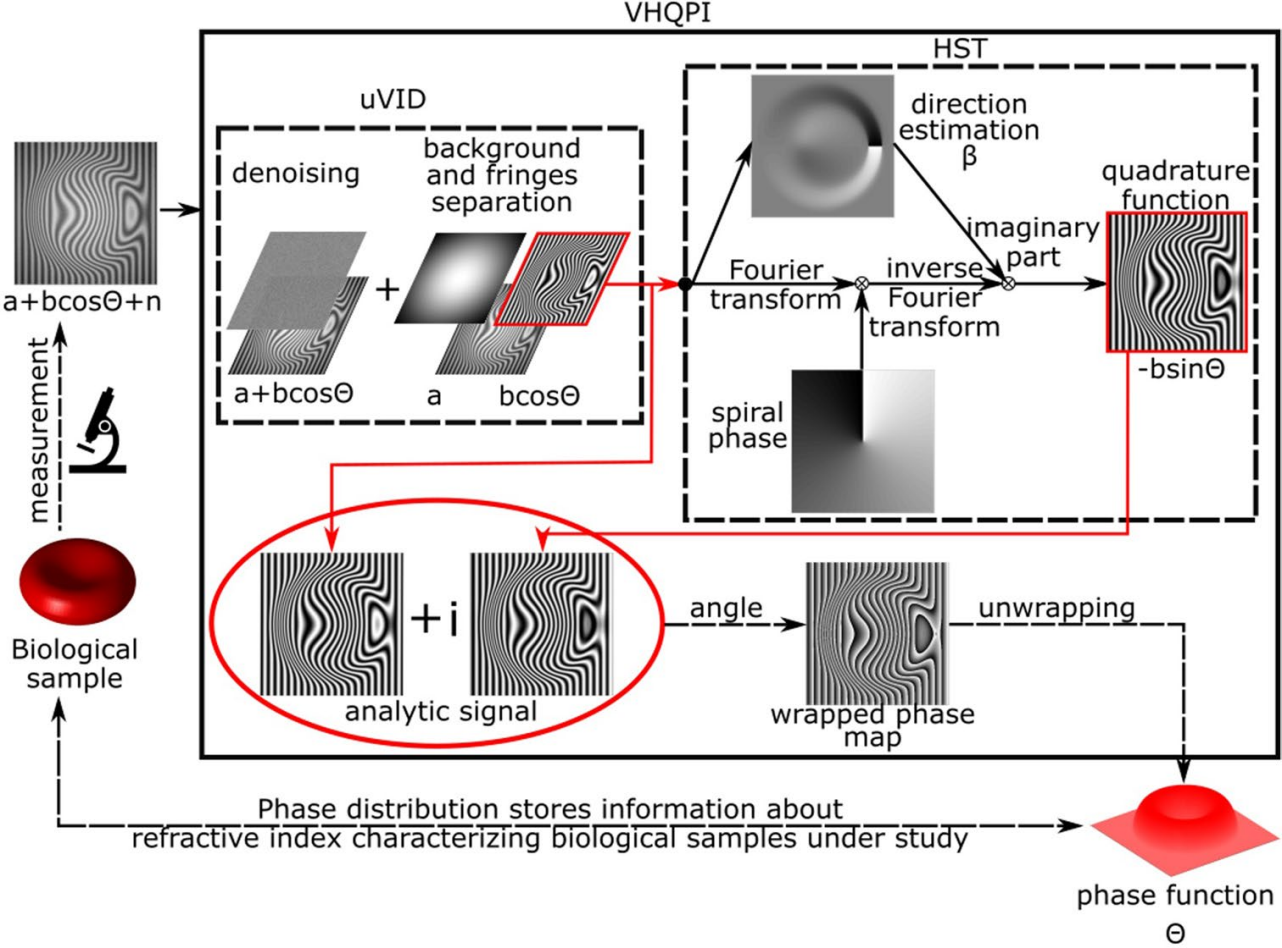
Working principle of the proposed VHQPI technique where the process starts on the left (biological sample measurement). Please see its detailed discussion in the text: unsupervised variational image decomposition (uVID) in “Interferogram initial filtering”, Hilbert spiral transform (HST) in “Filtered interferogram phase demodulation”.

The uVID-filtered noise-free and zero-mean-valued fringe term (minimized autocorrelation term in the Fourier domain) is then analyzed in the second step of the VHQPI using the Hilbert spiral transform (HST)^56,64^. It is important to emphasize that carrier-free single-shot interferogram analysis is a challenging fully 2D phase demodulation problem, whereas carrier-based Fourier transform phase demodulation is a 1D simplification^64^ of the Hilbert transform analytic relation. The HST therefore requires the local fringe direction map (β, modulo 2π). It is estimated in two step process: (1) combined gradient and plane-fitting method^65^ is employed for local fringe orientation map modulo π calculation, (2) further advanced filtered unwrapping follows to obtain modulo 2π direction map—β of interest. Basing on the pre-filtered interferogram and its local direction map the HST calculates quadrature component to the starting single-shot interferogram. In this way complex analytic fringe pattern, see Fig. 1, is generated with its real part defined as the uVID pre-filtered interferogram and imaginary part denoted as the HST generated quadrature fringes:

2 $$AFP = 2\sqrt {I_{1} I_{2} } \cos (\theta ) - i\exp ( - i\beta )F^{ - 1} \{ SPF*F[ 2\sqrt {I_{1} I_{2} } \cos (\theta )] \} ,$$

where SPF denotes spiral phase function and F denotes Fourier transform operator. Angle of this complex analytic fringe pattern constitutes the wrapped phase map of particular interest in QPI. After phase unwrapping^66^ and plane fitting for the residual linear term removal VHQPI returns high quality phase map quantifying sample-induced optical path delay which is directly related to the refractive index structure and/or thickness of the studied transparent bio-sample.

It is to be emphasized that in the demonstrated VHQPI approach the Hilbert spiral transform introduced by Larkin^64^ is used for phase demodulation/reconstruction purpose. It is a very capable approach, however, several requirements need to be fulfilled. Interferogram has to be of zero mean value—this is satisfied upon background term removal using the uVID approach. Moreover, amplitude term (*b* in Eq. 1) has to be a slowly varying function and generally low-pass banded with respect to the carrier cosine term. It is known as so-called Bedrosian theorem^67^ and is fulfilled for relatively low carrier spatial frequencies (slightly-off axis regime) for all pure phase objects and, e.g., in the time-averaged interference microscopy for MEMS vibration testing where amplitude modulation of the interferogram is described by the Bessel function and encodes the information on spatial distribution of the vibration amplitude^68,69^. However, the Bedrosian requirement is clearly not satisfied, e.g., in a very common imaging case of the well-established USAF target with sharp bars and numbers in it. Amplitude demodulation of such detail-rich objects is, by definition, out of the scope of the presented method working in slightly-off axis configuration. It is to be showcased that off-axis configurations yield correct amplitude demodulation due to increased carrier. Interestingly, the Kramers–Kronig method recently proposed in^53^ allows for such detail-rich amplitude map demodulation (amplitude imaging) in case of autocorrelation term overlapping with the cross-correlation terms (slightly off-axis configuration). Additionally it is worth to emphasize that 2D Hilbert spiral transform needs local fringe direction map to perform phase demodulation, especially in slightly-off axis and quasi on-axis configurations. For off-axis interferograms the phase retrieval can be simplified into a classical Fourier transform technique, which is a 1D representative of the 2D Hilbert spiral transform not taking account for the fringe local direction^64^.

#### Fringe direction map β calculation and its impact on the phase demodulation

Fringe orientation map of pre-filtered fringe pattern is estimated using the combined gradient and plane fitting approach^65^. It requires setting single parameter value—the size of the plane fitting window. In general, the smaller the window is the denser fringes could be analyzed (better resolution) but influence of the noise is stronger (deteriorating orientation map quality). The orientation map, which is calculated in modulo π form, answers the question whether interference fringes are locally horizontal or vertical. It needs to be ‘unwrapped’ to the modulo 2π fringe direction map, which in turn answers the question whether vertical fringes are facing right or left and horizontal fringes face up or down. We propose to use simple orientation unwrapping algorithm utilized before in phase unwrapping^70^. Unwrapped orientation map usually has its values spread over incidental range and modulo 2π operation is performed giving the final result of fringe direction map estimation (information, in every pixel, about the angle of the normal to the fringe and OX axis). To enhance the direction map quality we apply sine–cosine filtering resulting in smoothed and denoised map. It is crucial to avoid all artificially introduced direction discontinuities and noise which will result in erroneous jumps of wrapped phase fringes. Upon phase unwrapping those phase jumps will be the source of large disqualifying errors.

Proposed processing path is executed iteratively increasing the plane fitting window. After phase demodulation and phase unwrapping for each window size the gradient of the unwrapped phase map is calculated and its mean value stored. The window size with resulting minimum phase gradient mean value is the one ultimately chosen. This automatic and adaptive approach can be summarized in the following steps.

**Figure 2:**
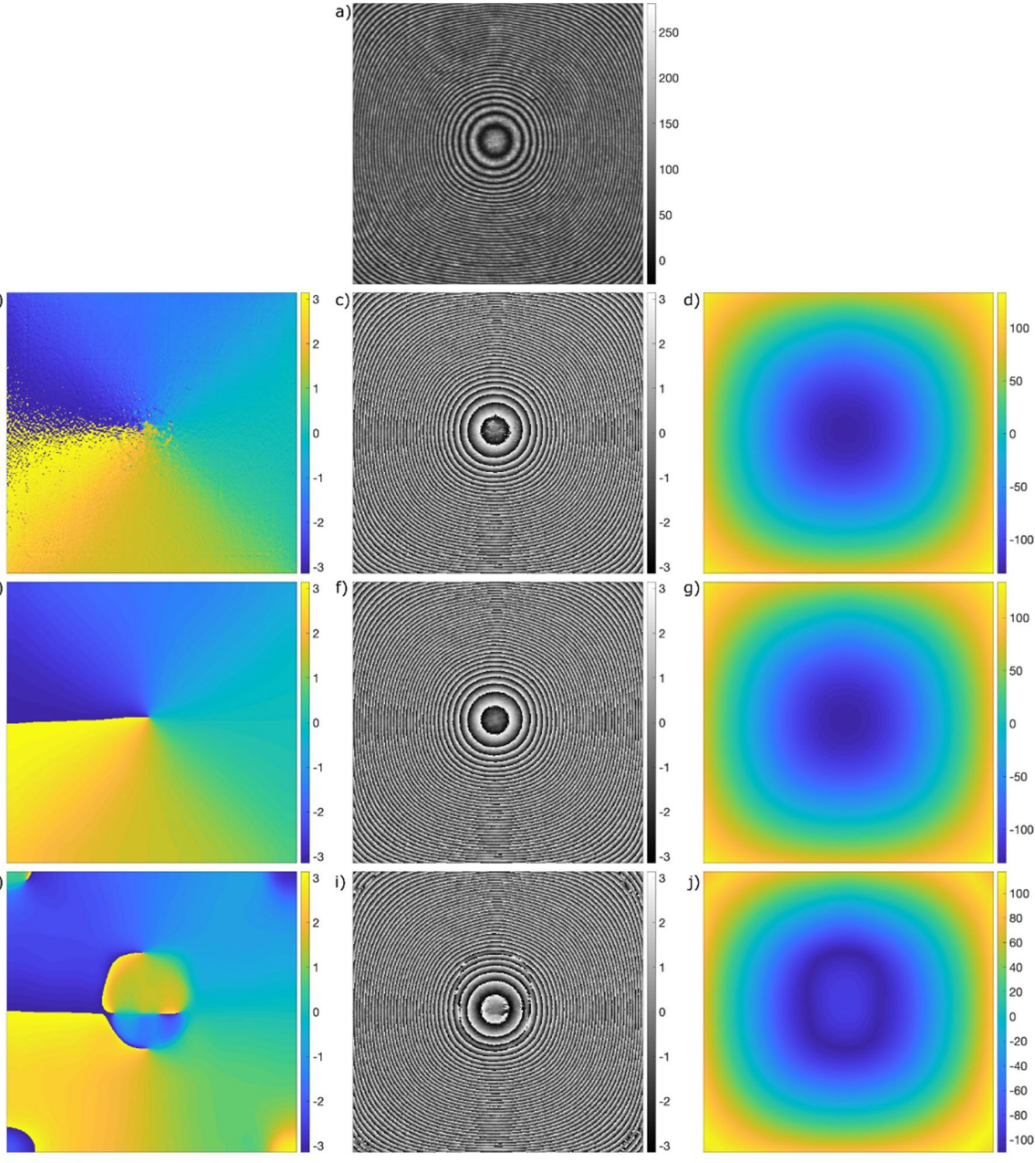
Studying the local fringe direction estimator and its influence on the phase demodulation quality: (**a**) experimental interferogram with closed fringes, (**b**) noisy direction map estimated using small window without sine–cosine filtering, (**c**) noisy VHQPI phase map demodulated using direction map (**b**), (**d**) unwrapped phase (**c**), (**e**) correct direction map estimated using small window and sine–cosine filtering, (**f**) correct VHQPI phase map demodulated using direction map (**e**), (**g**) correctly unwrapped phase (**f**), (**h**) incorrect direction map estimated using large window with sine–cosine filtering, please note artificial erroneous discontinuities present, (**i**) incorrect VHQPI phase map (with artificial phase jumps) demodulated using erroneous direction (**h**), (**j**) unwrapped correct phase (**i**).

- Step 1: set iteratively the window size *w* starting from *w* = 3 [3 × 3 pixels] to *w* = 13 [13 × 13 pixels] with increment of 2 pixels. Our studies shown that *w* = 13 is the optimal limit and there is no need to continue with the search due to erroneous orientation map estimation (larger windows induce artificial direction discontinuities, see Fig. 2h).
- Step 2: calculate the orientation map of pre-filtered interferogram using combined gradient and plane fitting method^65^ and pre-set window size [*w* × *w* pixels].
- Step 3: unwrap^70^ the orientation map to direction map with sine–cosine filtering applied to remove noise. Sine–cosine filtering is employed calculating sine and cosine of unwrapped orientation map and filtering both distributions by eliminating first three empirical modes calculated using the enhanced fast empirical mode decomposition (EFEMD) approach^71^. This step is finalized by obtaining direction map as arctangent of filtered sine term over filtered cosine term.
- Step 4: calculate phase map of the pre-filtered interferogram using estimated direction map.
- Step 5: compute mean gradient value of the unwrapped phase map and store its value.
- Step 6: track the minimal phase gradient value and use the corresponding window size employed for this phase map demodulation as the final window size selected and the phase map as the final calculated phase distribution. Minimization of the phase gradient serves the purpose of selecting smoothest phase map calculated using error-free direction estimate, as direction imperfections introduce phase jumps clearly detectable by gradient operation.

Figure 2 highlights the importance of the correct fringe direction map estimation. We use experimental interferogram with concentric closed fringes, Fig. 2a, due to its real-life features and full range of direction angles (0–2π).

We start with 3 × 3 window and no sine–cosine filtering approach: direction map is depicted in Fig. 2b. Corresponding phase map calculated using the Hilbert spiral transform employed to filtered interferogram guided by the estimated noisy direction map is presented in Fig. 2c. It is worthy to note the noise transferred from direction map to the phase map, especially in the central fringe region, as a consequence of the lack of a sine–cosine filtering. Unwrapped noisy phase map is depicted in Fig. 2d. We proceed with applying 3 × 3 window and sine–cosine filtering obtaining correct direction map, Fig. 2e and accurate phase demodulation result, Fig. 2f. Unwrapped correctly demodulated phase map is presented in Fig. 2g. When we increase the window size to 11 × 11 pixels erroneous orientation map, Fig. 2h, is produced. Although it is generally smooth (as a result of bigger window used) it contains artificial direction discontinuities introducing disqualifying errors to demodulated phase map modulo 2π, Fig. 2i. Those visible phase jumps will result in jeopardized unwrapping, Fig. 2j. It is readily observable comparing phase maps in Figs. 2g and 2j, which are unwrapped^66^  versions of correct wrapped phase map Fig. 2f and erroneous one Fig. 2i, respectively.

## Numerical evaluation using synthetic interferograms

**Figure 3:**
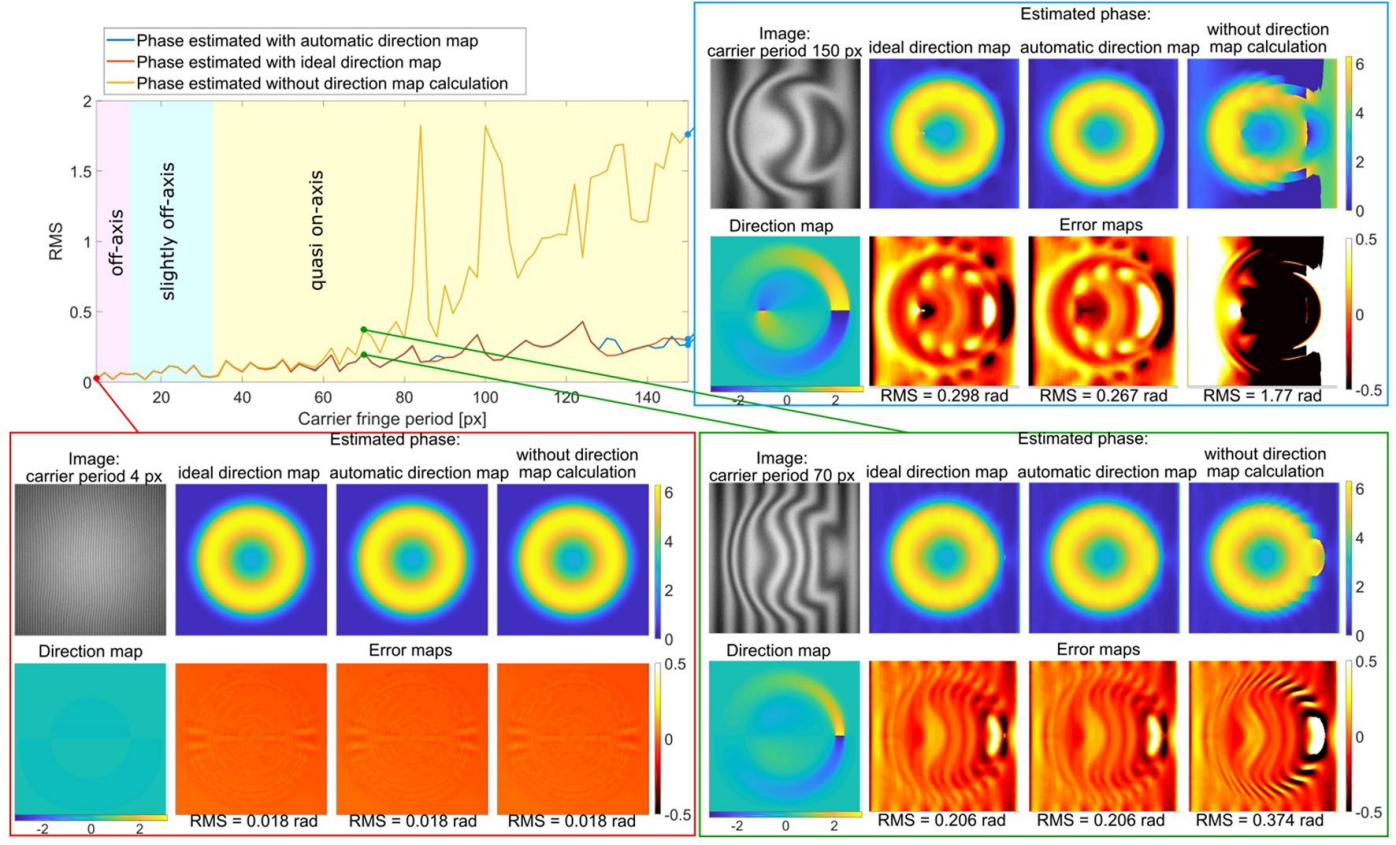
Performance evaluation of the proposed VHQPI method and influence of the direction map estimation on the phase accuracy. See Supplementary Visualization 1 for the full-range dynamic animation.

In this section, we are conducting the VHQPI performance evaluation using simulated interferograms. This numerical study aims at attempting to define the acceptable regime of eligible fringe carrier frequencies (number of fringes required). To do so we simulated a series of holograms with different carrier frequencies ranging from very few fringes in the image to the case of 4 pixels per period. Phase function used in this numerical experiment was simulated as a shape of the unstressed red blood cell with the use of the equation of Evans and Skalak^72^:

3 $$z = D_{0} \sqrt {1 - \frac{{4(x^{2} + y^{2} )}}{{D_{0}^{2} }}} \left( {a_{0} + \frac{{a_{1} (x^{2} + y^{2} )}}{{D_{0}^{2} }} + \frac{{a_{2} (x^{2} + y^{2} )^{2} }}{{D_{0}^{4} }}} \right),$$

where *D*_0_ = 7.82 μm denotes the average cell diameter, *a*_0_ = 0.0518, *a*_1_ = 2.0026 and *a*_2_ = − 4.491 are experimentally derived constants. Simulated fringe patterns series used during our analysis presented in Fig. 3 can be described as:

4 $$I(x,y) = a(x,y) + \cos \left( {5z + \frac{2\pi }{T}x} \right) + 0.05 \cdot randn(512),$$

where *x*, *y* denote image coordinates simulated here with the use of Matlab function *meshgrid*(*1:512*), *a*(*x*, *y*) denotes background simulated as Gauss function, *T* denotes fringe period varying in the range 4–150 px with the step value equal to 2 px. Additive Gaussian noise is simulated with the use of *randn*(*512*)*—*a Matlab function, which allows to simulate normally distributed pseudorandom numbers.

We numerically tested the proposed method in Fig. 3 generating and analyzing a full set of synthetic interferograms. To highlight the importance of the local fringe direction estimation, we compared the phase map results retrieved utilizing our proposed iterative algorithm with the ones obtained employing ideal numerically generated direction map and the ones calculated without the direction map estimation step. It can be noticed that for some range of carrier periods (high overall density of fringes) the results do not differ significantly between three studied approaches. However, with the increase of the carrier fringe pattern period value (decrease of the overall fringes density/spatial frequency), local fringe direction estimation starts to play increasingly important role in the whole phase calculation process. It can be said that as long as analyzed fringes are simple in the meaning of their shape (small deviation from straightness) the calculation of direction map is not necessary. On the other hand, in the case of more complex fringe patterns the results estimated without the direction map calculation are not acceptable. Moreover, the results calculated with the use of our algorithm do not differ from the ones calculated with the use of the ideal, numerically generated direction map. This fact validates the accuracy of the proposed numerical method both in terms of direction estimation and phase calculation for a wide-ranging shape/densities of fringes. It is important to emphasize that spectrum-overlapping is a natural effect of increasing fringe period. We have included Supplementary Visualization 1 with animation of full studied range of synthetic interferograms to highlight the efficiency, robustness, stability and versatility of the proposed VHQPI phase demodulator. Root mean squared error was calculated to quantitatively evaluate the algorithm. Promisingly low RMS values exhibited by the VHQPI over a large range of periods are to be emphasized, Fig. 3. It is worthy to note that appropriate estimation of the fringe direction map plays crucial role in the quasi on-axis regime, where 2D Hilbert spiral transform (and our proposed VHQPI scheme) is especially useful. In off-axis regime 2D Hilbert spiral transform can be simplified to direction-free Fourier transform calculations.

The VHQPI is highly data-driven and detail preserving (see Fig. 3 and experimental validation) technique due to local interferogram filtration using the uVID followed by local HST-based direction-aided fully 2D phase demodulation. This means that in the case of very dense fringes (high spatial frequency of the carrier, large inclination angle between interfering beams) when the sampling is not sufficient our algorithm can preserve the nonlinear characteristic of the undersampled fringes (they can result in periodic errors present in the output phase map^73,74^). The Fourier transform^37^, continuous wavelet transform^75,76^, or windowed Fourier transform^77^ are based on the harmonic functions and assuming cosinusoidal profile of the fringe they estimate phase more reliably in the case of extremely high carrier spatial frequency. The fully separated hologram spectrum is needed, however, which is not the case in VHQPI operating also in carrier-free mode (spectral overlapping). Nonetheless, the proposed VHQPI method is not recommendable in the case of very low carrier frequency combined with low phase delay introduced by the sample. Very few visible coherent fringes are then spatially overlapping with incoherent interferogram background and efficient decomposition/filtration is unfortunately impossible. It is worthy to note, however, that even in the case of the quasi on-axis hologram recording (i.e., blue frame in Fig. 3) and strong incoherent background we can successfully apply the VHQPI approach in carrier-free regime. It is also beneficial when studied object is introducing sufficient phase delay to generate fringes^24^.

## Experimental evaluation

The experimental validation of the proposed method is implemented using the embodiment of a BX60 Olympus microscope and employing the recently proposed technique named Spatially Multiplexed Interferometric Microscopy (SMIM)^78^. A superluminescent diode  (SLD from Exalos, Model EXS6501-B001, 10 mW of optical power, a 650 nm central wavelength, a 6 nm spectral bandwidth) illuminates the input plane, where the useful FOV is spatially multiplexed into object/sample and reference/clear regions. The objective (Olympus UMPlanF infinity corrected 20x 0.46NA) and the tube lens system magnify the input plane spatially multiplexed distribution at the output port of the microscope, where a charge-coupled device (CCD) camera (Basler A312f, 582 × 782, 8.3 μm px size, 12 bits/px) is placed. In this way, conventional (nonholographic) imaging is recorded, but a 1D diffraction grating (Ronchi ruled grating, 20 lp/mm period) is introduced in the analyzer insertion slot just before the tube lens to allow output plane replicas and, thus, interferometric recording. Grating lateral displacement realizes the phase-shifting sequence. Issues about proper selection of the 1D grating and details on the setup can be found in^78^. Importantly, proposed versatile numerical add-on for phase demodulation can be applied to wide range of optical setups, including other configurations, e.g., regular Mach–Zehnder or Michelson based DHM systems.

Proposed Variational Hilbert Quantitative Phase Imaging technique is experimentally validated using calibration target (90 μm polystyrene microsphere) and utilizing static and dynamic cell analysis. For static bio-examination prostate cancer cells (provided by the Fundación Instituto Valenciano de Oncología—FIVO) and red blood cells (provided by Proiser R + D S.L.) were selected. Dynamic evaluation is performed using live spermatozoon cells (provided by Proiser R + D S.L.). Reference techniques in terms of the Fourier transform (FT) and the Hilbert–Huang transform (H2PM) were deployed for the VHQPI benchmarking purpose. Quantitative evaluation in terms of root mean square calculation has been employed. To quantify and compare the noise robustness feature we calculated standard deviation of sample-free regions in the retrieved phase maps^79,80^. Different lines (PC-3 and RWPE-1) of prostate cells were prepared following the same procedure. The cells were cultured in RPMI 1640 medium with 10% fetal bovine serum, 100 U/ml penicillin and 0.1 μg/ml streptomycin at standard cell culture conditions (37 °C in 5% CO_2_ in a humidified incubator). Once the cells reach a confluent stage, they were released from the culture support and centrifuged. The supernatant fluid is discarded by centrifugation and the cells are resuspended in a cytopreservative solution and mounted on a microscope slide.

**Figure 4:**
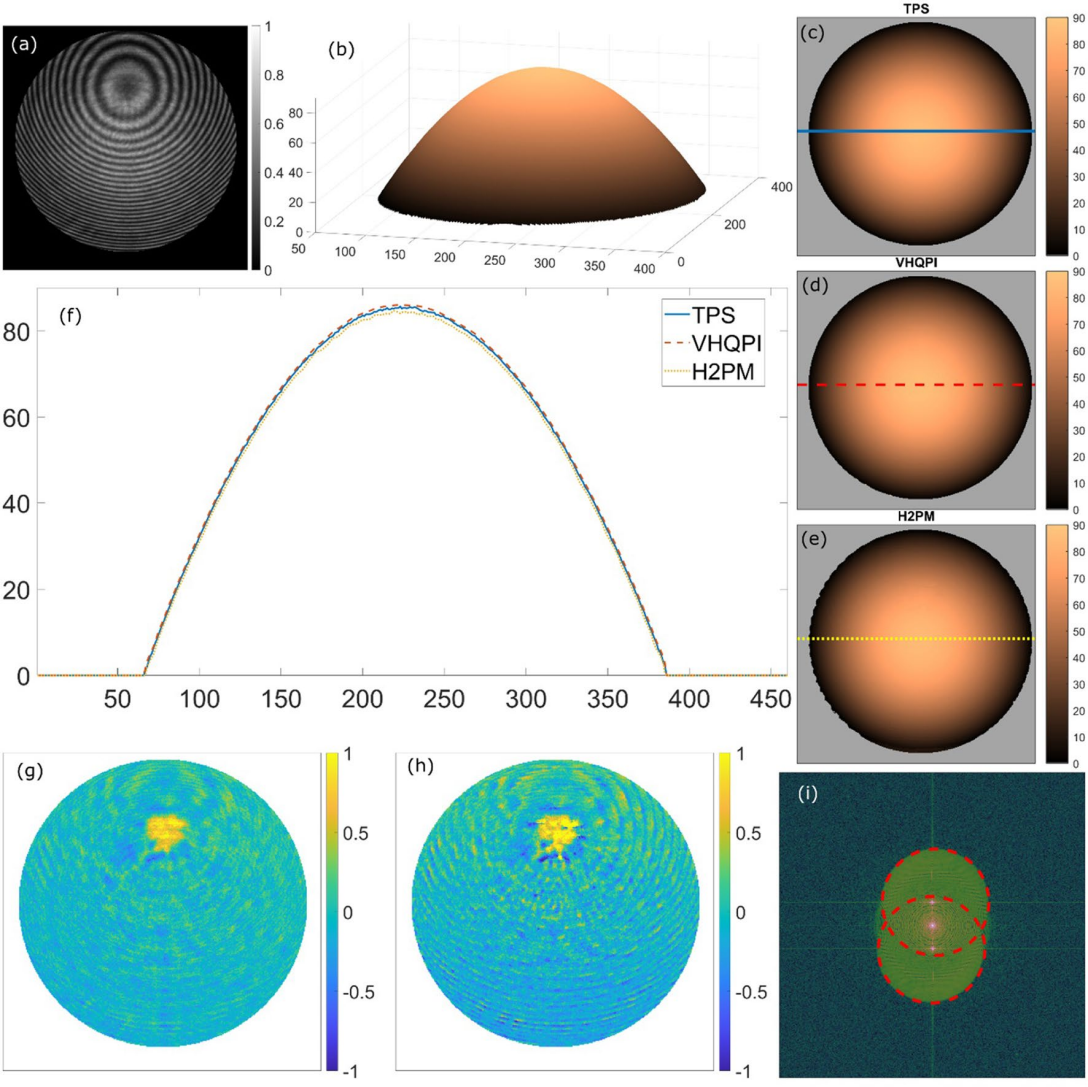
Phase imaging calibration test: (**a**) the microsphere interferogram, (**b**) 3D visualization and (**c**) 2D map of the reference phase distribution calculated using the temporal phase-shifting (TPS) method, (**d**) phase map derived employing the VHQPI method, (**e**) phase map obtained using the H2PM technique, (**f**) cross-sections through the bead phase profiles; phase error maps calculated in comparison with the phase-shifting ground truth for (**g**) the VHQPI and (**h**) the H2PM, and (**i**) the interferogram spectrum with indicated cross-correlation terms (red) significantly overlapping with each other and autocorrelation term.

Results presented in Fig. 4 consider the analysis of single polystyrene microsphere of approx. 90 μm in diameter immersed into water. We include this study as a calibration measure where a known sample is used to validate the capability of phase imaging provided by the proposed VHQPI technique. In Fig. 4a the interferogram is presented with masked-out microsphere-free area. The 5-frame temporal phase-shifting (TPS) technique was employed for calculating the reference ground truth phase profile—depicted in 3D (Fig. 4b) and 2D (Fig. 4c) afterwards. The phase map obtained using the proposed VHQPI method is presented in Fig. 4d, whereas the result of the H2PM phase demodulation is shown in Fig. 4e. As it can be readily observed in cross-section presented in Fig. 4f, both single-shot techniques, namely VHQPI and H2PM, ensure phase imaging capacity verified by the multi-frame temporal phase-shifting profile. Phase values in radians determined by the H2PM method are slightly lower, however. For further quantitative verification of both single-shot techniques the RMS values were calculated basing on the ground truth temporal phase shifting result. The VHQPI obtained 0.18 rad and outperformed the H2PM 0.24 rad error. Phase error maps of the VHQPI and H2PM are respectively presented in Figs. 4g and 4h, respectively, to facilitate localization of main error sources. For both methods central closed fringe is the most cumbersome area, which is characteristic for all single-shot phase demodulation techniques as in this ‘singularity’ fringe period approaches infinity (spatial frequency is close to zero), phase function has its global extremum and direction map has its vortex. It can be readily observed in Fig. 4h, that the H2PM method exhibits within whole bead area the fringe like phase error connected to the interferogram pre-filtering imperfections (non-zero mean value and non-cosinusoidal fringe profile). To sum up, the VHQPI is positively verified for phase imaging of calibration microsphere target, which corroborates its robustness to severely overlapped spectral cross-correlation terms, see the modulus of the interferogram’s Fourier transform in Fig. 4i with highlighted spectral apertures (cross-correlation terms in red). On an additional note, it is worthy to showcase that single-frame Fourier transform phase reconstruction is not applicable in this case due to the closed concentric fringes and fully overlapped spectrum.

**Figure 5:**
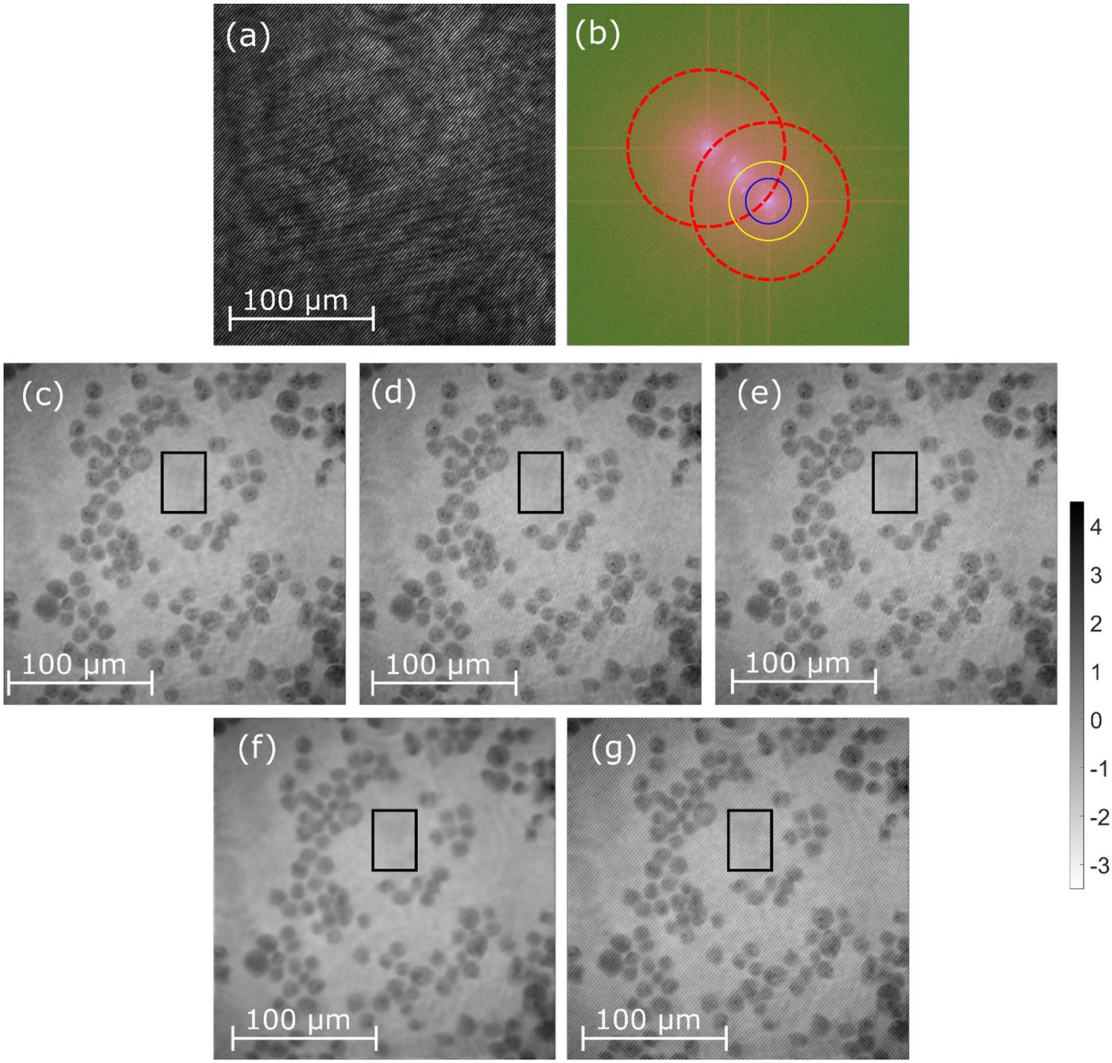
Fixed RWPE-1 bio-sample phase imaging: (**a**) the interferogram, (**b**) its spectrum with overlapping cross-correlation terms marked by red circles; phase demodulation results of (**c**) phase-shifting method, (**d**) the VHQPI, (**e**) the H2PM, (**f**) the FT with blue circle filtering aimed at minimizing fringe-like phase errors and (**g**) the FT with yellow circle filtering aimed at maximizing phase resolution. Black rectangles mark the sample-free areas selected for standard deviation calculation in noise suppression evaluation.

**Figure 6:**
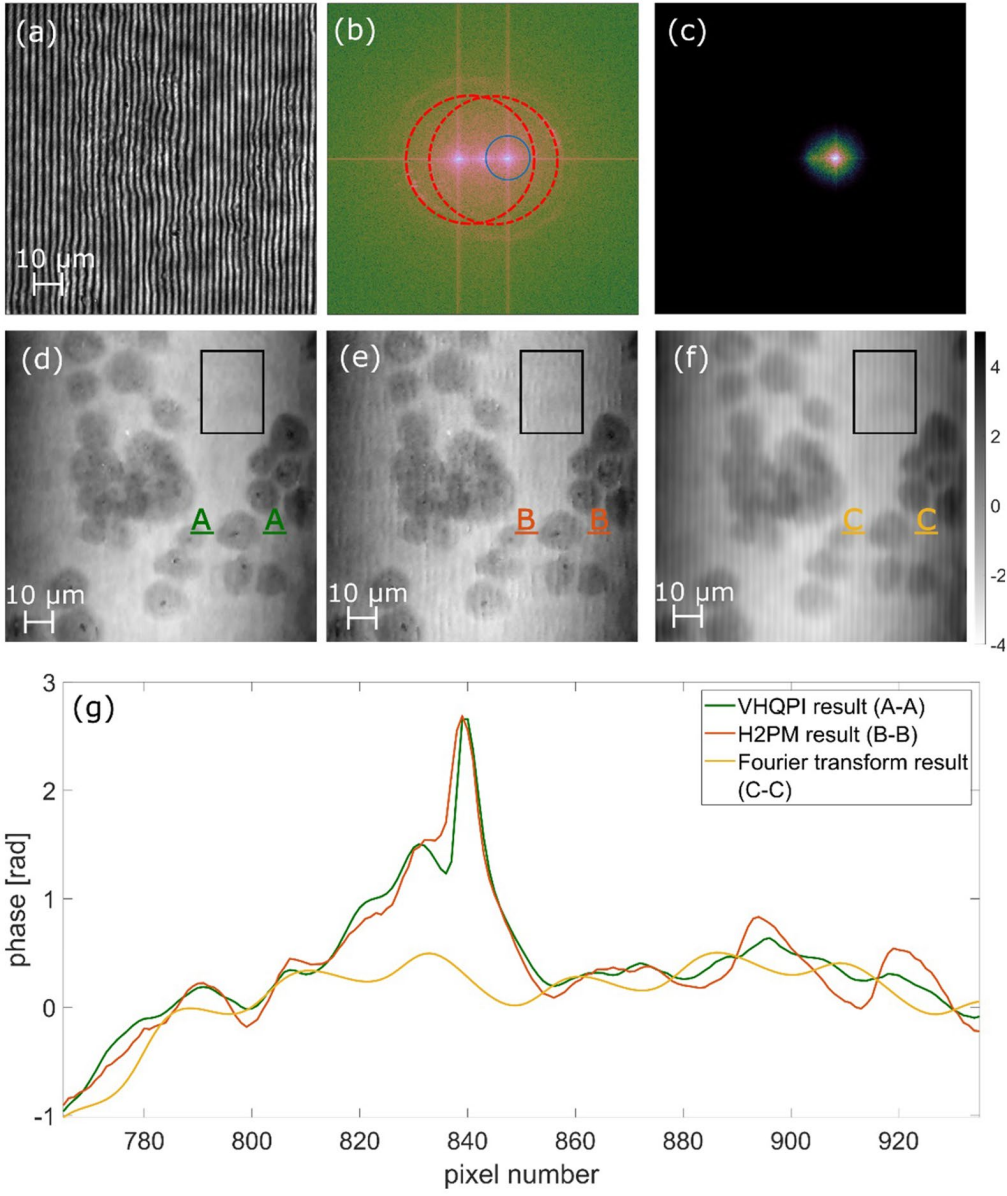
Prostate cancer PC-3 cells phase analysis: (**a**) the interferogram, (**b**) its FT with marked cross-correlation terms (in red dashed circles), (**c**) the optimal size of filtering window in the Fourier domain (corresponding to the blue circle in the FT), and (**d**)–(**f**) the results calculated using VHQPI, H2PM and FT, respectively (see Supplementary Visualization 2). Scale for (**d**)–(**f**) represents optical phase in radians. Cross-sections marked by green (*A*-*A* in VHQPI), red (*B*-*B* in H2PM) and orange (*C*-*C* in FT) dashed lines are presented at the bottom (**g**) showcasing detail-preservation feature of the proposed VHQPI. Noise suppression ability has been evaluated calculating standard deviations in marked rectangular sample-free areas: FT 0.19 rad, HHT 0.21 rad, VHQPI 0.17 rad.

**Figure 7:**
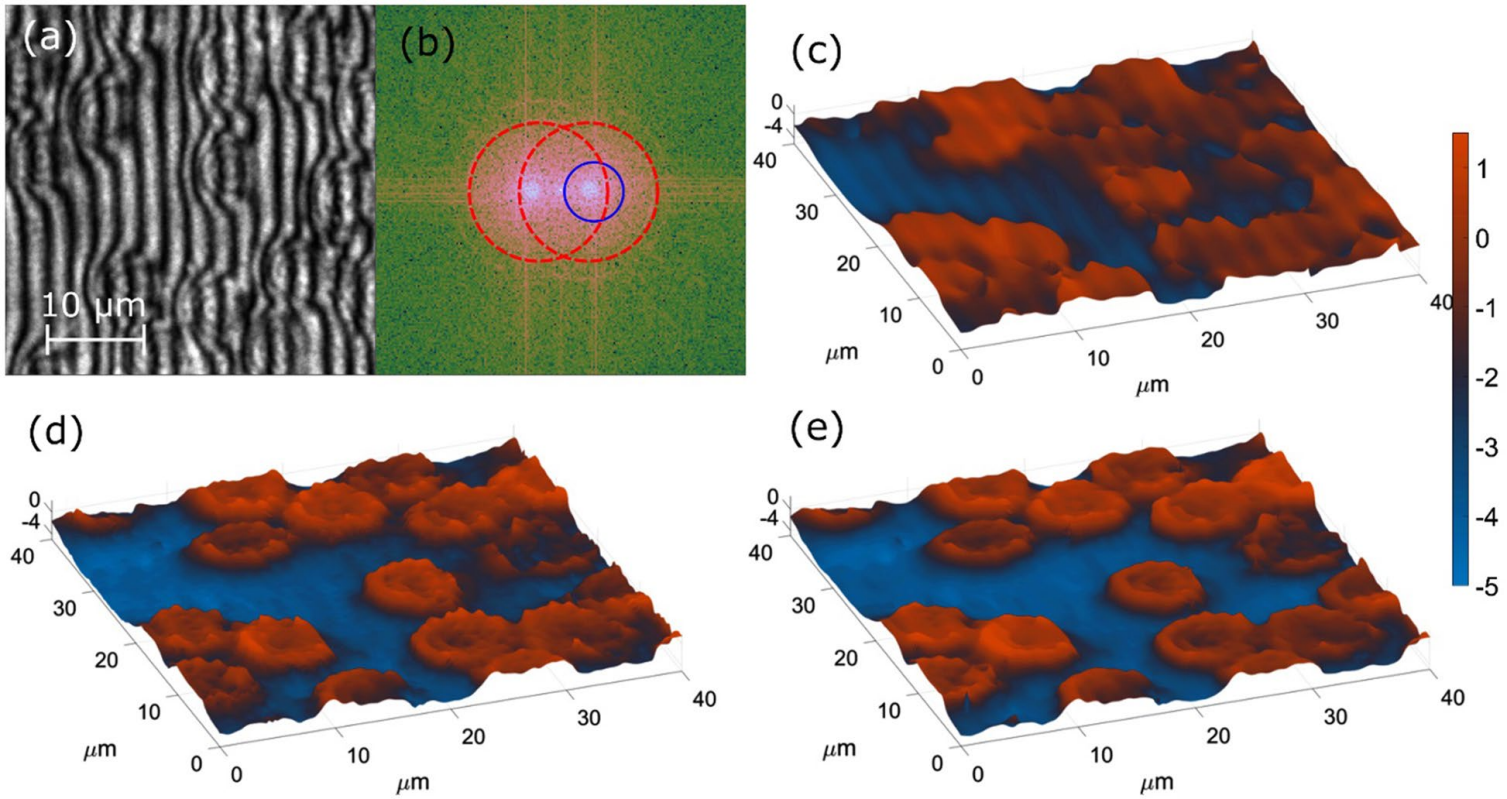
Degenerated red blood cells phase analysis: (**a**) the hologram (interferogram), its (**b**) spectrum and results of (**c**) FT, (**d**) H2PM and (**e**) VHQPI approaches. Lateral scale represents optical phase in radians for all QPI images. Interferogram spectrum is presented to highlight cross-correlation terms overlapping; additionally blue circle indicates Fourier filtering mask.

We continue the evaluation with fixed bio-samples phase imaging. Static RWPE-1 cells are analyzed in Fig. 5, static prostate cancer PC-3 cells are examined in Fig. 6, while static degenerated red blood cells are studied in Fig. 7. Recorded interferograms are presented in Figs. 5a, 6a and 7a, whereas the VHQPI results are demonstrated in Figs. 5d, 6d and 7e, respectively. The VHQPI is compared with reference single-shot phase analysis techniques: the Hilbert–Huang phase microscopy H2PM^24^ method (Figs. 5e, 6e, 7d) and the Fourier Transform FT^37^ approach (Figs. 5f, g, 6f, 7c).

In Fig. 5 the analysis concerns RWPE-1 cell line. Interferogram presented in Fig. 5a is of overall low spatial carrier frequency, which results in spectral overlapping of cross-correlation terms Fig. 5b. It is important to showcase that this configuration no longer fits under the scope of the slightly off-axis regime, thus it is termed as quasi on-axis, as carrier fringes are clearly visible and we are still relatively far from fully on-axis recording. The ground truth reference phase map was calculated using the phase-shifting method, Fig. 5c. Single frame phase retrieval outcomes are presented in Fig. 5d for the VHQPI, Fig. 5e for the H2PM, and Figs. 5f, and 5g for the FT. We have considered two cases of FT analysis marked with blue and yellow circles in spectrum, Fig. 5b. Phase calculated with blue circle filtering, Fig. 5f, is aimed at minimizing fringe-like phase errors and phase demodulation with yellow circle filtering, Fig. 5g, is focused on maximizing the phase resolution. The trade-off in Fourier Transform retrieval is clearly observable—increasing the filter size to enclose more object spatial frequencies we end up in introducing fringe like spectral overlapping error; decreasing the filter size to get rid of the fringe like phase error we end up severely limiting phase resolution. The VHQPI is free of such a trade-off, Fig. 5d, and the same conclusion is valid for efficiently implemented H2PM method (with correct filtering), Fig. 5e. It is important to note that parasitic interferences present in the optical setup will result in spurious sets of fringes, and they in turn will impact the phase distribution of single-shot processing, please observe additional periodic phase modulations in Figs 5d and 5e. It is to be stressed here that this is not linked with fringe like phase errors exhibited by the FT method, Fig. 5g, as those fringes closely mimic the interference ones from the interferogram intensity distribution, Fig. 5a. Parasitic interference fringe error is not visible in TPS phase map as a result of multi-frame phase-shifted interferogram subtraction. Quantitative assessment of overall accuracy of phase demodulation has been performed utilizing the phase-shifting map as the ground truth and reads: VHQPI RMS = 0.12 rad (e) HHT RMS = 0.13 rad, FT (blue filter) RMS = 0.15 rad, FT (yellow filter) RMS = 0.37 rad. Presented values corroborate accuracy of the proposed VHQPI technique. Noise suppression ability has been validated calculating standard deviation values within sample-free phase background areas: phase-shifting STD = 0.15, VHQPI STD = 0.16, H2PM STD = 0.17, FT (blue) STD = 0.12, FT (yellow) STD = 0.33.

**Figure 8:**
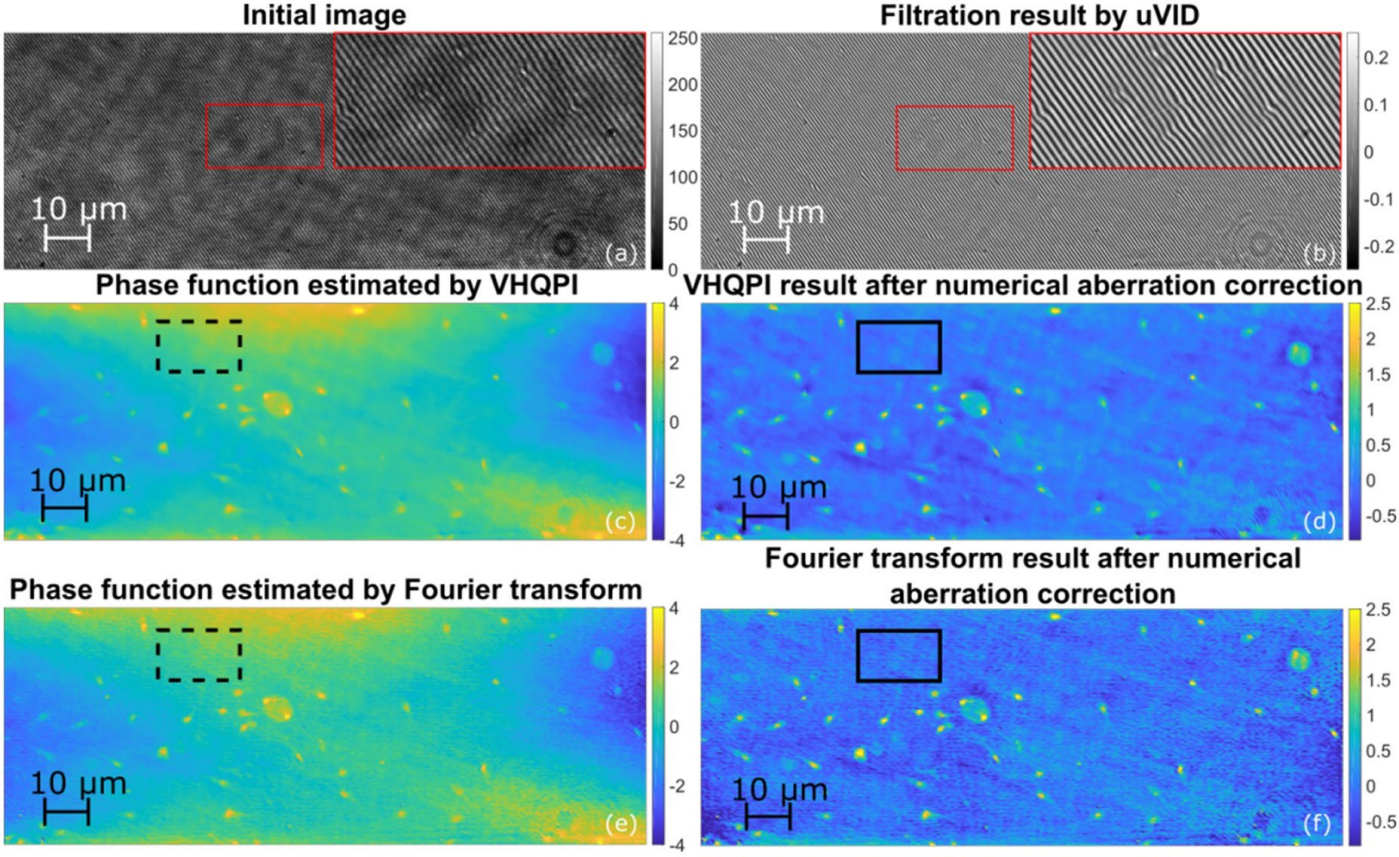
Spermatozoon dynamic phase analysis: (**a**) initial hologram, (**b**) its filtration by VHQPI, (**c**) phase function estimated by VHQPI, (**d**) VHQPI result after aberration correction, (**e**) phase function estimated by FT before, and (**f**) after aberration correction. See Supplementary Visualization 3 for full dynamic scene. Scale represents optical phase in radians. Rectangles indicate regions selected for aberration correction and noise suppression evaluation.

In Fig. 6 we have included the cross-section to visualize cell-detail-preservation feature exhibited by the proposed VHQPI method and showcase strong carrier-related limitations imposed by the FT approach. We have highlighted the influence of spectral filtering window size used in the FT—Supplementary Visualization 2 presents phase maps obtained for different increasing window sizes. Generally, window size is to be set manually to cover as much object spectral information as possible but to exclude the autocorrelation term, as when it is introduced it damages the phase analysis. Recently deep learning algorithm^81^ was introduced to enable efficient automatic FT filtering, but it does not help when spectral overlapping is encountered due to insufficiently high carrier spatial frequency versus rich object spectrum (the case studied in our experiments). In Figs. 6, 7, 8 FT phase maps were calculated using optimal window sizes. Nonetheless, in Figs. 6f and 7c, FT phase map lacks important object details and exhibits fringe-like error.

In the H2PM, the hologram pre-filtering is performed using empirical mode decomposition (EMD) approach. Large number of decomposition components (modes) generally increases the risk of losing valuable information upon manual or automatized filtering—reconstruction of the fringe term by summing up chosen modes or their selected areas. Moreover, noise removal employed by eliminating first mode (containing locally highest spatial frequencies of decomposed interferogram) is often not efficient as first mode tends to store information also about the high spatial frequency details of object (and dense fringes as well). It is to be emphasized that empirical mode decomposition itself is data-driven and depends on the extrema distribution of the analyzed interferogram—this fact also increases the risk of augmented filtering imperfections as extrema distribution is noise-driven. Mentioned reconstruction errors can introduce small but noticeable nonlinearities to fringe profile resulting in fringe-like phase analysis errors, see Fig. 6e and cross-sections in Fig. 6g. On the other hand, when executed correctly on valid image the H2PM is a vital local-detail-preserving phase analysis method, see Fig. 5e.

Figure 7 presents the analysis of the degenerated red blood cells (RBC), cumbersome due to their shape deterioration and flattening, especially when recorded with low carrier spatial frequency, Fig. 7a. Both FT, Fig. 7b, and H2PM, Fig. 7c, are not able to accurately image phase details and examine correctly RBC structure, although the H2PM compares favorably. The proposed VHQPI technique showing unique robustness skillfully enabled detailed phase imaging, Fig. 7d. Standard deviations of the phase background calculated for the VHQPI 0.13 rad, the H2PM 0.2 rad and the FT 0.23 rad provide quantitative metric of phase imaging quality promoting the proposed VHQPI.

Comparing with the H2PM technique, the proposed VHQPI algorithmic solution automatically and fully adaptively dissects fringe component as image texture with no need for fringe reconstruction nor introducing any parameters. Additionally, it successfully removes noise from interferogram preserving object related details, which is a characteristic feature of block-matching 3D filtering approach^55–57^. Moreover, the VHQPI employs automatic procedure to generate error-minimized fringe direction map and uses it to boost quality of the phase demodulation. In a consequence the VHQPI is remarkably versatile similarly to emerging deep learning approaches^82–85^ with the crucial difference that it requires no training at all and the result, i.e., Figs. 4d, 5c, 6d and 7e, is just one click away basing on the notion of default purely numerical add-on module. It can potentially aid a suite of modern fringe-pattern-based QPI techniques including DHM, ODT, HPM, DPM etc.

Experimental corroboration is continued using dynamic phase analysis of live spermatozoon, as shown in Fig. 8. It is associated with Supplementary Visualization 3 showing characteristic movement and distribution of cells. We have recorded the interferometric sequence using fully off-axis approach to facilitate reference FT phase retrieval. It can be noted that VHQPI result is corroborated by the reference FT technique and it is smoother than the reference with no significant phase detail ullage.

Due to the optical setup instrumental imperfections, retrieved phase maps can generally exhibit structured errors. In our case phase distribution is considerably affected by the astigmatism related phase-background (in both FT, Fig. 8c, and VHQPI, Fig. 8e). We propose novel post-processing phase-enhancement approach based on already introduced unsupervised Variational Image Decomposition (uVID). This time not interferogram but unwrapped phase map is decomposed and phase background (decomposition structure) is removed. Results of this new straightforward phase-map-driven aberration correction approach are presented in Fig. 8d for the FT and Fig. 8f for the VHQPI.

For quantitative analysis we have selected a region without spermatozoon—it is highlighted with dashed line rectangle in case without phase correction, Figs. 8c and 8e, and with solid black rectangle in case after proposed numerical phase correction, Figs. 8d and 8f. Mean values calculated within the marked sample-free areas dropped from 1.16 to 0.01 rad in VHQPI case, and from 1.11 to − 0.04 rad for the FT demodulation, after performing the numerical correction. Ideally mean value for aberration free phase map (in sample-free areas) would be close to zero, and with this regard numerical uVID phase correction can be seen as verified. We have also performed reference experimental phase correction procedure calculating the phase background as average over whole dynamic sequence of spermatozoon movement (Fig. 8, Supplementary Visualization 3). It serves the purpose very well as phase values within marked sample-free areas were not disturbed by spermatozoon movement. Experimental phase correction yields very good mean values equal to − 0.001 rad for the VHQPI and 0.002 for the FT, corroborating the proposed uVID-based numerical phase correction procedure.

In noise suppression verification process standard deviation values were calculated for marked sample-free regions yielding.

- for FT: 0.32 rad before aberration correction, 0.17 rad after numerical correction and 0.03 after experimental correction,
- for VHQPI: 0.30 rad before aberration correction, 0.09 rad after numerical correction and 0.01 after experimental correction.

These values constitute quantitative metric promoting the VHQPI in terms of lower phase reconstruction noise level.

The VHQPI method needs further acceleration of computational execution time, which scales with local period variations (spatial carrier frequency), to improve towards on-line quantitative phase map retrieval. In current implementation we focused on the disruptive versatility, robustness and accuracy of this single-frame numerical phase demodulator with a tradeoff in processing time which is around 15 s for off-axis, 150 s for slightly off-axis and 300 s for quasi on-axis 512 × 512 pixels simulated interferogram, on a low-cost PC (2.6 GHz processor and 16 GB RAM).

## Conclusions

Employing the QPI methodology in biomedical practice one can easily encounter samples (cumbersome ones resulting in too noisy, dim and complicated interferograms) and scenarios (dynamic events, strong local time dependent phase variations etc.) not fitting within the scope of a given QPI unit. We believe that the proposed Variational Hilbert Quantitative Phase Imaging solves those restrictions and broadens the applicability of QPI modules possibly enabling new results and mechanistic understandings. The VHQPI is reported as a versatile, accurate and robust numerical phase map demodulator. By employing the VHQPI purely as a numerical add-on module, one can enhance capabilities of a given QPI unit with no hardware modifications by, e.g.,

1. facilitating dynamic single-shot object characterization without the need for phase-shifting sequence recording and
2. enabling examination of complicated phase objects containing high frequency phase details in QPI layouts with insufficient spectral separation (overlapping of cross-correlation and auto-correlation spectral terms in slightly off-axis and quasi on-axis configurations).

First feature is instrumental in real-life biosensing, where characterization of dynamic phenomena is indispensable. Second feature is especially important when (1) decreased temporal/spatial coherence of the light source is used to minimize speckle noise, which can be followed by critical limit in recordable carrier frequency, and/or (2) large numerical-apertures are used to increase the lateral resolution of the phase imaging, which can be followed by significant overlapping of cross-correlation and auto-correlation terms in the interferogram spectrum (Supplementary Video 1).

It is vital to note that there are single-frame fringe analysis algorithms^86–91^ reported to be able to surpass the overlapping spectrum limitation but they were only applied to well defined (polynomial-based) slowly varying continuous phase functions. In QPI the objective is to study detail-rich highly diversified bio-samples; as every cell is different it is not possible to model all of them universally and retrieve correct detail-preserved phase map in optimization based manner^86–91^. Single-shot versatility of the VHQPI, understood as ability to accurately retrieve phase from single interferogram regardless the cell specificity and variability, is a considerable advantage and novelty in the QPI field. Moreover, it is to be emphasized that the VHQPI is user friendly and does not require any special tailoring nor specified operator skills. The VHQPI can be seen as numerically advanced extremely versatile and easy-to-use tool aiding any interferometric setup focused on QPI. Interferogram analysis is indispensable in fringe-based phase imaging and the VHQPI is designed to numerically solve these optical information encoding–decoding problems. It is scalable in terms of QPI method and object under test. Its potentially seamless application to non-biological object evaluation is evident.

The presented analysis provides a unique look into importance of the interference pattern phase demodulation for QPI, while the VHQPI expands its capabilities. Proposed novel computational strategy constitutes a step forward in addressing quantitative phase reconstruction challenges possibly enabling new results and elaborated understandings.

## Supplementary information

Supplementary Visualization 1.

Supplementary Visualization 2.

Supplementary Visualization 3.
