# Author Correction: Variational Hilbert Quantitative Phase Imaging

**DOI:** 10.1038/s41598-020-77491-3

**Published:** 2020-12-04

**Authors:** Maciej Trusiak, Maria Cywińska, Vicente Micó, José Ángel Picazo-Bueno, Chao Zuo, Piotr Zdańkowski, Krzysztof Patorski

**Affiliations:** 1 Institute of Micromechanics and Photonics, Warsaw University of Technology, 8 Sw. A. Boboli St., 02‑525 Warsaw, Poland; 2 Departamento de Óptica Y de Optometría Y Ciencias de La Visión, Facultad de Física, Universitat de Valencia, C/Doctor Moliner 50, 46100 Burjassot, Spain; 3 Jiangsu Key Laboratory of Spectral Imaging and Intelligence Sense, Nanjing University of Science and Technology, Nanjing, 210094 Jiangsu China

Correction to: *Scientific Reports* 10.1038/s41598-020-69717-1, published online 18 August 2020

The original version of this Article contained errors in the spelling of the authors Maria Cywińska, José Ángel Picazo-Bueno, Vicente Micó and Piotr Zdańkowski, which were incorrectly given as Maria Cywinska, Jose-Angel Picazo-Bueno, Vicente Mico and Piotr Zdankowski.

Furthermore, the Acknowledgements section was incomplete.

“We would like to thank Prof. Bjorn Kemper for fruitful discussions. We acknowledge the support of Faculty of Mechatronics Warsaw University of Technology statutory funds (Dean’s grant). Codes for VHQPI demodulation will be made available upon reasonable request. The study leading to the published manuscript has beed funded, in parts, by the National Science Center Poland (NCN) (2017/25/B/ST7/02049), Polish National Agency for Academic Exchange (PPN/BEK/2018/1/00511), Spanish Ministerio de Economía, Industria y Competitividad and the Fondo Europeo de Desarrollo Regional (FIS2017-89748-P), National Natural Science Foundation of China (61722506), Outstanding Youth Foundation of Jiangsu Province (BK20170034).”

now reads:

“We would like to thank Prof. Bjorn Kemper for fruitful discussions. We acknowledge the support of Faculty of Mechatronics Warsaw University of Technology statutory funds (Dean’s grant). Codes for VHQPI demodulation will be made available upon reasonable request. The study leading to the published manuscript has beed funded, in parts, by the National Science Center Poland (NCN) (2017/25/B/ST7/02049), Polish National Agency for Academic Exchange (PPN/BEK/2018/1/00511), Spanish Ministerio de Economía, Industria y Competitividad and the Fondo Europeo de Desarrollo Regional (FIS2017-89748-P), National Natural Science Foundation of China (61722506), Outstanding Youth Foundation of Jiangsu Province (BK20170034). Studies were funded by FOTECH-1 project granted by Warsaw University of Technology under the program Excellence Initiative: Research University (ID-UB).”

These errors have now been corrected in the PDF and HTML versions of the Article.

